# Supplementary material for: Microfluidic characterisation reveals broad range of SARS-CoV-2 antibody affinity in human plasma
Source: Life Sci Alliance. 2021 Nov 30;5(2):e202101270. doi: 10.26508/lsa.202101270 (PMC8645332; doi:10.26508/lsa.202101270)
Supplement: Supplementary file 1 [file LSA-2021-01270_TableS1.docx]

**Table S1**. Demographic characterisation of the individuals used in our study.

| Cohort | Total number | Number male | Number female | Number undefined | Median age (IQR) - years |
| --- | --- | --- | --- | --- | --- |
| Convalescent individuals | 19 | 19 | 0 | 0 | 27 (22-49) |
| Healthy blood donors | 20 | 8 | 10 | 2 | 31 (24-45) |
| Hospital patients | 3 | 2 | 1 | 0 | 67 (65-74) |
