# Supplementary material for: Microfluidic characterisation reveals broad range of SARS-CoV-2 antibody affinity in human plasma
Source: Life Sci Alliance. 2021 Nov 30;5(2):e202101270. doi: 10.26508/lsa.202101270 (PMC8645332; doi:10.26508/lsa.202101270)
Supplement: Supplementary file 2 [file LSA-2021-01270_TableS2.docx]

**Table S2**: Comparison of outcome on ACE2 competition and cytopathic-effect based assay, for all samples which were tested in the ACE2 competition, cytopathic-effect based assay, and had low enough background autofluorescence to be assessed by MAAP. Positive means that the radius has decreased compared to the ACE2-S1 complex. This is the aggregate statistics of the results presented in Fig. S7. A positive result in the ACE2 competition assay is here defined as yielding an effective R_h_ value smaller than 5.6 nm.

|  |  | Cytopathic-effect based neutralisation assay | |  |
| --- | --- | --- | --- | --- |
|  |  | Positive | Negative | Total |
| ACE2 competition | Positive | 29 | 1 | 30 |
|  | Negative | 2 | 4 | 6 |
|  | Total | 31 | 5 | 36 |
