# Supplementary material for: Microfluidic characterisation reveals broad range of SARS-CoV-2 antibody affinity in human plasma
Source: Life Sci Alliance. 2021 Nov 30;5(2):e202101270. doi: 10.26508/lsa.202101270 (PMC8645332; doi:10.26508/lsa.202101270)
Supplement: Supplementary file 3 [file LSA-2021-01270_TableS3.docx]

**Table S3**: Comparison of outcome on ACE2 competition and cytopathic-effect based neutralisation assay. Here, we show the hydrodynamic radius based on the ACE2 competition assay as well as the titre of the neutralisation assay. Sera which did not neutralise in the cell plaque assay at any of the titres tested are indicated by ‘none’, whereas those that were not tested are indicated by ‘N/A’.

| \| Patient ID \| ACE2 competition assay R_h_ /nm \| Critical titre for the cytopathic-effect based neutralisation assay (fraction) \| \| --- \| --- \| --- \| \| 1 \| 4.83 \| 0.0125 \| \| 2 \| 4.98 \| 0.0125 \| \| 3 \| 4.86 \| 0.0125 \| \| 4 \| 5.36 \| 0.003125 \| \| 5 \| 4.84 \| 0.0125 \| \| 6 \| 4.79 \| 0.05 \| \| 7 \| 4.82 \| 0.05 \| \| 8 \| 4.62 \| 0.05 \| \| 9 \| N/A \| 0.003125 \| \| 10 \| 5.14 \| 0.0125 \| \| 11 \| 5.11 \| 0.0125 \| \| 12 \| 4.98 \| 0.003125 \| \| 13 \| 5.18 \| 0.05 \| \| 14 \| NA \| 0.05 \| \| 15 \| 5.06 \| 0.05 \| \| 16 \| 5.25 \| 0.05 \| \| 17 \| 5.76 \| none \| \| 18 \| 5.16 \| 0.05 \| \| 19 \| 5.91 \| none \| \| 20 \| 5.64 \| none \| \| 21 \| 5.67 \| 0.05 \| \| 22 \| 4.74 \| 0.05 \| \| 23 \| 5.14 \| 0.05 \| \| 24 \| N/A \| 0.05 \| \| 25 \| 5.79 \| 0.0125 \| \| 26 \| 4.84 \| 0.05 \| \| 27 \| 5.41 \| 0.0125 \| \| 28 \| 5.07 \| 0.05 \| \| 29 \| 4.77 \| 0.003125 \| \| 30 \| 5.35 \| 0.05 \| \| 31 \| 5.26 \| 0.05 \| \| 32 \| 4.81 \| 0.05 \| \| 33 \| 4.72 \| 0.05 \| \| 34 \| 5.67 \| none \| \| 35 \| 4.96 \| 0.0125 \| \| 36 \| 5.51 \| 0.05 \| \| 37 \| 5.12 \| none \| \| 38 \| N/A \| none \| \| 39 \| 5.30 \| 0.0125 \| \| 40 \| 5.08 \| 0.05 \| | |
| --- | --- | --- | --- | --- | --- | --- | --- | --- | --- | --- | --- | --- | --- | --- | --- | --- | --- | --- | --- | --- | --- | --- | --- | --- | --- | --- | --- | --- | --- | --- | --- | --- | --- | --- | --- | --- | --- | --- | --- | --- | --- | --- | --- | --- | --- | --- | --- | --- | --- | --- | --- | --- | --- | --- | --- | --- | --- | --- | --- | --- | --- | --- | --- | --- | --- | --- | --- | --- | --- | --- | --- | --- | --- | --- | --- | --- | --- | --- | --- | --- | --- | --- | --- | --- | --- | --- | --- | --- | --- | --- | --- | --- | --- | --- | --- | --- | --- | --- | --- | --- | --- | --- | --- | --- | --- | --- | --- | --- | --- | --- | --- | --- | --- | --- | --- | --- | --- | --- | --- | --- | --- | --- | --- | --- |
|  |  |
